# Implications of existing local (mal)adaptations for ecological forecasting under environmental change

**DOI:** 10.1111/eva.12840

**Published:** 2019-07-30

**Authors:** Richard J. Walters, David Berger

**Affiliations:** ^1^ Centre for Environmental and Climate Research Lund University Lund Sweden; ^2^ School of Biological Sciences University of Reading Reading UK; ^3^ Department of Ecology and Genetics, Evolutionary Biology Centre Uppsala University Uppsala Sweden

**Keywords:** adaptation, assisted migration, climate change, conservation biology, ecological forecasting, evolutionary rescue, gene flow

## Abstract

Standing genetic variation represents a genetic load on population fitness but can also support a rapid response to short‐term environmental change, and the greatest potential source of such standing genetic variation typically exists among locally adapted populations living along an environmental gradient. Here, we develop a spatially explicit simulation model to quantify the contribution of existing genetic variation arising from migration–mutation–selection–drift balance to time to extinction under environmental change. Simulations reveal that local adaptation across a species range associated with an underlying environmental gradient could extend time to extinction by nearly threefold irrespective of the rate of environmental change. The potential for preadapted alleles to increase the rate of adaptation changes the relative importance of established extinction risk factors; in particular, it reduced the importance of the breadth of environmental tolerance and it increased the relative importance of fecundity. Although migration of preadapted alleles generally increased persistence time, it decreased it at rates of environmental change close to the critical rate of change by creating a population bottleneck, which ultimately limited the rate at which de novo mutations could arise. An analysis of the extinction dynamics further revealed that one consequence of gene flow is the potential to maximize population growth rate in at least part of the species range, which is likely to have consequences for forecasting the consequences of ecological interactions. Our study shows that predictions of persistence time change fundamentally when existing local adaptations are explicitly taken into account, underscoring the need to preserve and manage genetic diversity.

## 1 INTRODUCTION

The speed at which species can adapt to environmental change and reduce the associated risk of extinction depends in large part upon how much standing genetic variation exists to support a response (Gonzales, Ronce, Ferriere, & Hochberg, 2013; Lynch & Lande, 1993). This variation, while advantageous in a dynamic environment, imposes a genetic load on population fitness in more predictable or steady environments (Pélabon, Armbruster, Hansen, Bolstad, & Pérez‐Barrales, 2012). Given the amount of genetic variation is itself subject to optimization (Lande & Shannon, 1996), it is important to define maladaptation in relative versus absolute terms since the latter has the potential to cause mutational meltdown and extinction (Baake & Gabriel, 2000). Nonetheless, what constitutes a maladaptation even in absolute terms in one part of a species range could still be considered adaptive in another. Understanding how these two opposing effects of segregating variation affects evolutionary potential and extinction risk of natural spatially structured populations is one of the fundamental contemporary challenges in conservation genetics and evolutionary forecasting (Brady, 2019; Derry, 2019).

Predictive models of ecological responses to environmental change, so‐called “species distribution models” (SDMs), often miss several biological mechanisms known to influence extinction risk, notably the role of evolutionary potential and existing genetic differentiation (Botkin et al., 2007; Fordham, Brook, Moritz, & Nogués‐Bravo, 2014; Urban et al., 2016; Walters, Blanckenhorn, & Berger, 2012). Ecological forecasts that fail to account for the possibility of adaptation are arguably unduly pessimistic since all that is required for adaptation to occur is trait variation, heritability and selection (Skelly et al., 2007). This is true both at low rates of environmental change, when mutation rate can sustain evolutionary change under directional selection (Bürger & Lynch, 1995; Hoffmann & Sgrò, 2011; Lindsey, Gallie, Taylor, & Kerr, 2013; Lynch & Lande, 1993), and at higher rates of change during which standing genetic variation alone can sustain a short‐ to mid‐term adaptive response (Barrett & Schluter, 2008). Under anthropogenic climate change, the fear is that the rate of change will be too great for all but the most numerous, fecund and short‐lived organisms to successfully adapt to Lindsey et al. (2013), while the hope is that evolution can rescue those populations subject to a limited environmental shift (Gonzalez et al., 2013).

This last decade has seen a vast improvement in predictions of species persistence and the case for evolutionary rescue with more complex models replacing SDMs. These models attempt to incorporate eco‐evolutionary dynamics into demographic predictions (so‐called “dynamic eco‐evolutionary models”, DEEMs), and recent years have seen continuous advances with focus on spatially explicit modelling and explorations of the effects of gene flow on adaptive rates among interconnected demes. Indeed, the most beneficial contribution to standing genetic variation, and thus the initial response to selection, will likely come from those populations already locally adapted to an underlying environmental gradient associated with the driving force of selection (Jump & Penuelas, 2005). Local adaptation has long been considered ubiquitous (Endler, 1986), though it is not often rigorously demonstrated (Blanquart, Kaltz, Nuismer, & Gandon, 2013; Kawecki & Ebert, 2004). Meta‐analyses reveal it is only evident in ca. 70% of studies (Hereford, 2009; Leimu & Fischer, 2008). There is however clear evidence that clines in fitness optima with latitude and elevation are widespread among organisms as diverse in life history as protists (Boenigk, Jost, Stoeck, & Garstecki, 2007), dung flies (Blanckenhorn et al., 2018) and trees (Rehfeldt et al., 1999), suggesting there is ample potential to adapt to predicted changes in temperature and precipitation (De Carvalho et al., 2010; Visser, 2008). Moreover, the rapid re‐establishment of such clines following species invasions demonstrates that the process can be facilitated primarily from existing genetic variation (Blanckenhorn et al., 2018; Gilchrist et al., 2008; Hoffmann & Weeks, 2007). Nevertheless, under climate change genotypes will face the additional challenge of intraspecific competition as they traverse occupied habitat, which may impact the success of management policies that aim to promote habitat connectivity or directly assist in the migration of genotypes (Aitken & Whitlock, 2013).

Recent spatially explicit models provide insights into the dynamics of adaptation at range margins (e.g. Bourne et al., 2014; Gilbert et al., 2017) and the intricate role of gene flow in either promoting or impeding adaptation. These complex dynamics have also been demonstrated in recent empirical studies utilizing either very clever experimental set‐ups (e.g. Bell & Gonzalez, 2011; Bosshard et al., 2017) or rare and highly informative cases in the wild (e.g. Bolnick & Nosil, 2007; Fitzpatrick, 2019). Given the multifarious effects of gene flow on rate of adaptation highlighted by both past and these more recent models (reviewed in: Garant, Forde, & Hendry, 2007; Legrand et al., 2017; Lenormand, 2002; Thuiller et al., 2013), there is a need for mechanistic DEEMs to better understand and predict the consequences of complex interactions between ecological and evolutionary processes across space (Ehrlén & Morris, 2015) and the factors that ultimately determine species range limits (Polechová et al., 2015). Indeed, recent examples applied to fruit flies (Bush et al., 2016) and alpine plants (Cotto et al., 2017) have demonstrated the utility of such approaches by showing how predictions of evolutionary demography across spatially explicit landscapes can change fundamentally compared to predictions of less informed models.

With the exceptions of humans, no species truly has a global distribution (Gaston & Fuller, 2009) and the finding that populations situated at the range limits lack genetic variance in some species has been interpreted as evidence of a fundamental constraint that could prevent adaptation to predicted environmental change (Gaston, 2003; Kellermann, Heerwaarden, Hoffmann, & Sgrò, 2011; Magiafoglou & Hoffmann, 2003). Nonetheless, this empirical finding is also consistent with the theoretical prediction that maladaptation arises at range limits due to “gene swamping” associated with migration of alleles from elsewhere in the species range that reduces the efficacy of selection relative to genetic drift (Kirkpatrick & Barton, 1997; Polechová, Barton, & Marion, 2009; 2015). However, if this latter explanation is correct, the implication is that range limits are not constrained by a lack of sufficient adaptive standing genetic variation and that a mere disruption to gene flow, for example due to a vicariant event (Garcia‐Ramos & Kirkpatrick, 2006) or change in interspecific competition (Case & Taper, 2000), could result in rapid evolutionary change. While empirical evidence supports the prediction that migration load associated with asymmetric gene flow can substantially impede local adaptation (e.g. Bolnick & Nosil, 2007), the extent to which it ultimately limits range size and adaptive responses to environmental change remains hotly debated (Connallon & Sgrò, 2018; see also Bridle, (2019 ) in this special issue).

In this paper, we present novel findings from a spatially explicit allelic simulation model run on virtually generated landscapes to investigate how local adaptation affects predictions of mean time to extinction under various rates of environmental change. By modelling genetic variance as an emergent property of selection efficiency and dispersal rate along an environmental gradient, we (a) quantify the relative contributions of de novo mutation and standing genetic variation to extinction risk within a given habitat, (b) demonstrate how local adaptation to an environmental gradient can exacerbate or moderate established risk factors and (c) identify potential temporal and spatial signals of impending extinction. We discuss the implications of our findings with respect to conservation management of maladapted meta‐populations.

## 2 METHODS

### 2.1 Simulation model overview

A spatially explicit individual‐based model was programmed in C++ (Borland Builder Studio files are available from the corresponding author upon reasonable request; the simulation data that support the findings of this study are openly available in Dryad Digital Repository at http://doi.org/[doi]) to simulate rate of adaptation and time to extinction with respect to a variable rate of environmental change across virtual landscapes. These landscapes were defined by varying levels of habitat fragmentation and steepness of the environmental gradient across its range. The model is based on the quantitative genetic principles of the Lynch and Lande (1993) model. Individual fitness is a function of the environment, represented by a Gaussian distribution with mode, *z*, and breadth, *ω*, to define the optimal environmental conditions and tolerance to environmental variation, respectively (Lynch & Gabriel, 1987). Here, we consider how *z* responds to directional selection as a single quantitative trait when additive genetic variance is an emergent property of migration, mutation, selection and drift across complex landscapes generated using a fractal algorithm. The mismatch between the mode *z* of the environmental tolerance curve and the mean environmental conditions encountered by any one generation determines the strength of fecundity selection on our virtual species, which is sexual, monandrous, self‐incompatible and has discrete generations. The model proceeds in five steps: birth, local population regulation, dispersal, mating and death. We then extend the model to investigate the roles of environmental tolerance breadth, population regulation, female fecundity and the number of loci encoding the breeding value ***ź***, the genetic contribution to the fitness function defining the optimal environmental conditions for each individual. To attribute the contribution of de novo mutation per se to adaptation rate and extinction times, we also compare the results from three different models: (a) a complete evolutionary model in which both standing genetic variation and mutation in ***ź*** contribute to an adaptive response; (b) a null model in which an adaptive response is only possible due to standing genetic variation; and (c) a second null model that assumes observed phenotypic variance in *z* has no heritable basis.

### 2.2 Virtual species life history and ecology

The life cycle of the model organism is as follows: birth, dispersal, mating and death, with simulated demographic stochasticity in survival to reproduction, dispersal distance, number of offspring and sex ratio. The number of females recruited to each patch is drawn from a binomial distribution (*N_t_* = total adults, *p* = .5, where number of males = *N_t_* – number of females), limiting the number of reproducing parents to the sex with the fewest individuals, thereby introducing an Allee effect. The number of offspring produced per female at the stage of reproduction is drawn from a Poisson distribution, Pois ($\lambda =2B_{\text{opt}}$), where $B_{\text{opt}}$ is the maximum mean fecundity per capita of an individual in its optimum environment (*z* = *θ*). For a given mismatch between the phenotype and its environment (z≠θ), fecundity declines as a Gaussian function:

(1) $$B_{z,t}=B_{\text{opt}}\cdot \exp\left[-\frac{{\left(z-\theta_{t}\right)}^{2}}{2\omega^{2}}\right],$$

where *ω* ^2^ represents the width of the environmental tolerance curve, a variable inversely related to the strength of selection (Lynch & Lande, 1993). A large value of *ω* ^2^ indicates that the species is an environmental generalist subject to relatively weak stabilizing selection (Bürger & Krall, 2004).

Population regulation of demes is imposed using the density‐dependent model of Maynard Smith and Slatkin (1973):

(2) $$N_{t+1}=\frac{{\overset{¯}{B}}_{t}N_{t}}{1+\left({\overset{¯}{B}}_{t}-1\right){\left(N_{t}/K_{S}\right)}^{c}},$$

where $N_{t}$ is adult population size at time *t*, ${\overset{¯}{B}}_{t}$ is mean reproductive rate per capita at time *t* (i.e. $R_{o}$), $K_{S}$ is the carrying capacity of the deme, and *c* is a parameter describing the form of competition, driving the dynamics of populations. The form of competition described by parameter *c* varies from contest to scramble, producing under‐compensatory ( *c* < 1), over‐compensatory (1<c<2) and chaotic (*c* > 2) dynamics, where *c* = 1 represents logistic population growth. This particular model of density dependence is known for its flexibility and success in describing empirical data (Bellows 1981). For our simulations, $N_{t+1}$ out of a possible ${\overset{¯}{B}}_{t}N_{t}$ offspring were randomly assigned to the following generation, except in cases where $N_{t+1}$ > ${\overset{¯}{B}}_{t}N_{t}$(e.g. when intrinsic rate of increase *r* < 1), in which case all offspring were recruited.

Dispersal distance, *d*, of adult individuals was modelled as a negative exponential distribution from the centre of a grid cell as:

(3) $$d=\frac{\ln\left(1-x\right)}{-1/\overset{¯}{d}},$$

where $\overset{¯}{d}$ is the mean dispersal distance and *x* is a random number between 0 and 1 drawn from a uniform distribution. The value of $\overset{¯}{d}$ was standardized to a constant 2.5% of range size, which is comparable to empirical observations for mobile vertebrates (see García‐Ramos & Kirkpatrick, 1997). Direction of movement was drawn at random from a uniform distribution. When the polar coordinate (*d*, *Ф*) placed the individual beyond its current habitat cell and into another, the individual was reassigned to the new deme, and when it was placed into a nonhabitat cell, the individual was assumed to die and was removed from the population.

### 2.3 Genetics

Diploid individuals carry two alleles for each of *m* unlinked loci ranging from 3 to 50. Together, their additive value determines the breeding value ***ź*** of the mode of the environmental tolerance curve. The phenotypic value of the mode ***z*** is then derived from the Gaussian distribution N(*µ* = ***ź***, $\sigma^{2}=V_{E}$), where $V_{E}$ is the environmental variance standardized to a value of 1. We omit epistasis and dominance effects from our model since it has been shown that the exclusion of dominance and epistasis from such models has little to no negative effects on the efficacy of predictions (Crow, 2010). Additive genetic variance in our model is therefore simply equal to total genetic variance (i.e. $V_{A}=V_{G})$, and narrow‐sense heritability is equal to: *h* ^2^ = $V_{G}$/$V_{P}$ (noting that phenotypic variance, $V_{P}=V_{G}+V_{E}$(Lynch & Walsh, 1998)). The strength of stabilizing selection against breeding values is equal to $V_{S}=\omega^{2}+V_{E}$, yielding estimates of $V_{S}/V_{P}$ ranging from around 1 to 200 in accordance with empirical estimates of quadratic selection differentials (Johnson & Barton, 2005; Kingsolver et al., 2001). Following Bürger and Lynch (1995), we set genic mutation rate per generation, *µ*, to 2 × 10^–4^ so that for a species with 10 unlinked loci, the genomic mutation rate, 2*mµ*, was equal to 0.004. Mutational effect, *α* ^2^, was randomly drawn from the Gaussian distribution $N(\mu =0,\sigma^{2}=0.25)$ giving a total mutational input, $V_{M}/V_{E}$, of 0.001, which is consistent with estimates from empirical studies (Lynch & Walsh, 1998). Mutational effect, *α* ^2^, was weighted accordingly for different number of loci, *m*, in order to maintain a constant $V_{M}/V_{E}$, that is multiplied by 10/*m*. Here, we implement a “continuum‐of‐allele” model, where the mutational effect is added to the value of the existing allele.

To assess the extent to which genetic differentiation among demes was attributable to genetic drift versus adaptation, we also modelled evolution at an additional biallelic neutral locus subject to an equivalent mutation rate (2*µ*), causing the allele value to change from 1 to 0 or vice versa. We then calculated two standard fixation indices: *F*_ST_, a measure of allele frequency disequilibrium for neutral genetic markers:

(4) $$F_{\text{ST}}=\frac{\text{var}\left(p_{\text{o}}\right)}{p_{\text{o}}\left(1-p_{\text{o}}\right)},$$

where $p_{o}$ is the frequency of allele *p* for a biallelic locus within a subpopulation (Wright's original derivation, 1951); and *Q*_ST_, an analogous measure for quantitative traits:

(5) $$Q_{\text{ST}}=\frac{V_{A,\text{among}}}{V_{A,\text{among}}+2V_{A,\text{within}}},$$

where $V_{A,\text{within}}$ is the mean within‐deme additive genetic variance. The ratio of these two measures is traditionally used to infer the extent of local adaptation. When *Q*_ST_ > *F*_ST_, there is evidence for diversifying selection (and local adaptation), when *Q*_ST_ < *F*_ST_, there is evidence for stabilizing selection, and when *Q*_ST_ ≈ *F*_ST_, genetic differentiation can be attributed to random genetic drift (Whitlock, 2008). During simulations, calculations of $V_{A,\text{within}}$ were conducted for all demes with ten or more individuals. To evaluate the potential role of habitat fragmentation on genetic diversity per se, we also calculated total allelic richness (*A*, the number of unique alleles) across the species range.

To better understand the contribution of de novo mutation to evolutionary rescue, we conducted an additional analysis on a subset of simulation runs to follow the fate of all mutations under directional selection for 100 generations after they naturally arise in order to identify success factors associated with persistence and final allele frequency.

### 2.4 Landscape replicate generation

Patterns of habitat fragmentation were generated using the midpoint displacement algorithm by Saupe (1988). The fractal algorithm produces three‐dimensional landscapes characterized by two key parameters, namely the spatial autocorrelation (Hurst exponent *H*, which varies from 0 to 1) and the variance in displacement of points (*σ* ^2^). The Hurst exponent increases the “roughness” of the surface, such that the fractal dimension is equal to 3-H. Increasing *H* from 0 to 1 creates a gradient in the level of habitat fragmentation from high to low. The algorithm has been well documented and tested as a method to produce realistic “neutral” landscapes (Hargrove, Hoffman, & Schwartz, 2002; With, 1997) suitable to assess the impacts of habitat fragmentation on extinction risk (With, 2004; With & King, 1999) and species diversity (Körner & Jeltsch, 2008). For our simulations, we iterated the algorithm six times to produce a grid (2^6^ + 1)^2^ in size and then “flooded” the landscape to retain the arbitrary number of 500 suitable habitat patches (cells) comprising 11.85% of the total grid size (see Figure S1 for examples). To avoid artefacts associated with edge effects, all generated landscapes with more than 1 cell bordering the edge of the grid were rejected.

These landscapes represent suitable habitat patches for reproduction, which could be thought of as islands in an archipelago, forest fragments in an agricultural landscape or the distribution of host plants. Along one side of this landscape, we impose an environmental gradient, which could be thought of as a climatic gradient associated with latitude or altitude. To avoid confounding effects of habitat fragmentation with the (possible) extent of local adaptation, we standardized the steepness of this environmental gradient *b* to range size (and therefore dispersal rate) (cf. parameter *b* ^*^; Kirkpatrick & Barton, 1997). Accordingly, we consider *b* to have standardized units of $\theta {\overset{¯}{d}}^{-1}\omega^{-1}$, which can be used as a measure of selective pressure experienced across the landscape.

### 2.5 Extinction dynamics

For a subset of simulation runs, the temporal dynamics of extinction were monitored at the deme level to reveal how relative fitness, population size and habitat occupation changed in space. Here “relative fitness” is a proportional measure calculated with respect to the maximum possible fitness value that could be attained when the mode of the individual fitness function matches the environmental conditions, i.e. when ***z*** = *θ*). In addition, the fate of novel mutations arising under directional selection was tracked for 100 generations to identify to what extent persistence time and increases in allele frequency could be explained by the mutation effect size, allele effect size, genotype–environment mismatch, place of origin as distance along the environmental gradient and time of origin.

### 2.6 Initialization of simulations

Each habitat cell of the landscape was initialized with a local carrying capacity $K_{D}$ equal to 1/500 of total carrying capacity *K_T_*. For our simulations, $K_{D}$ varied between 10 and 100 individuals. For all scenarios, alleles were randomly initialized with one of five values ranging from −0.4 to 0.4 (in 0.2 increments) giving a mean genotype of zero. To ensure equivalent allelic variance among simulations when using a different number of loci, these initial values were weighted by √(50/loci number), where 50 represents the maximum number of unlinked loci used in simulations. Migration–mutation–selection–drift balance was given time to establish during a burn‐in phase of 20,000 generations prior to simulation runs. To standardize initial conditions for each landscape configuration, each scenario started with the same equilibrated genetic data saved from the burn‐in phase. To ensure randomization, all nonuniform pseudorandom numbers were generated using the Mersenne Twister algorithm (open source code: http://www.agner.org/random), individuals within demes were always accessed in random order, and mating, reproduction and population regulation were processed for all demes before dispersal was initiated.

Environmental conditions, *θ*, at the centre of the species range (x=0) were initially set to a mean of zero and a variance of one. An environmental gradient was then imposed along one dimension of the landscape such that mean local environmental conditions were equal to: θ+bx, where *b* is the change in environmental conditions experienced per grid cell. Two extreme scenarios were considered to illustrate how segregating genetic variation arising from gene flow between locally adapted demes contributes to persistence under environmental change. In the first scenario, populations are locally adapted to the same environment across the entire landscape (i.e. b=0), while in the second scenario, populations are locally adapted to an environmental gradient that is just steep enough to maintain an equivalent population size and site occupancy over a given landscape (i.e. *b* ≫ 0). Hereon, we refer to scenario 1 as “no environmental gradient” and scenario two as “steep environmental gradient”.

Rate of environmental change, *k*, was modelled as a linear function of time *t* with variance *ϵ*, such that *θ_t_* = *kt* + *ϵ*. During simulations, environmental change was initiated at rate *k* for up to 50,000 generations or until extinction occurred. If populations persisted for 50,000 generations for any one simulation run, all results for the particular rate of change *k* were discarded to avoid a truncated measure of mean time to extinction. In general, populations surviving 10,000 generations were likely to persist indefinitely (greater than 100,000 generations). The critical rate of change *k_c_*, the rate of environmental change at which the population can still just maintain a positive population growth rate, that is λ=1 (Lynch & Lande, 1993), was estimated here by incrementally decreasing *k* in 0.01 steps until time to extinction exceeded 50,000 generations. For each combination of parameters, we conducted 100 simulations, and to assess the effect of habitat fragmentation, we further replicated these simulations across 50 to 100 uniquely generated landscapes of a given Hurst value. Unless otherwise stated, parameter values were set to those listed in Table 1.

**Table 1:** List of variables used in the model and their parameterization

Character	Variable	Notation	Parameter range used here
Landscape	Hurst exponent	*H*	0–1
	Variance in displacement of points	$\sigma^{2}$	200
	Total carrying capacity	$K_{T}$	5,000–50,000
	Subpopulation carrying capacity	$K_{S}$	0.002*K*
	Environmental gradient	b	variable
Environment	Environmental conditions with time	$\theta_{t}$	variable
	Rate of environmental change generation^−1^	k	0.01–1
	Variance in environmental conditions	ϵ	1
Life history	Mode of the environmental tolerance curve	z	variable
	Breeding value of z	***ź***	variable
	Breadth of the environmental tolerance curve	ω	2.5–20
		$\omega^{2}$	6.25–400
	Subpopulation size of adults, with time	$N_{t}$	variable
	Maximum fecundity per capita	$B_{\text{opt}}$	5 to 100
	Form of density‐dependent regulation	*c*	0.5–3
	Mean dispersal distance	$\overset{¯}{d}$	0.025 × range size
	Dispersal distance of an individual	d	variable
Genetics	Phenotypic variance	$V_{P}$	variable
	Genetic variance	$V_{G}$	variable
	Environmental variance	$V_{E}$	1
	Mutation rate per generation	μ	2 × 10^−4^
	Number of loci	*m*	3–50
	Mutational effect	$\alpha^{2}$	0.25

## 3 RESULTS

To investigate the relative contribution of existing local (mal)adaptations to persistence under directional selection, we simulated two alternative scenarios in which we might expect a minimum versus maximum amount of standing genetic variance to accumulate among populations across the species range. In the first scenario, the total amount of standing genetic variation maintained across the landscape reflects local adaptation to a single environmental mean. This scenario serves as an evolutionary null model that is equivalent to a spatially explicit approximation to past analytical models developed for populations of a finite size (e.g. Burger & Lynch, 1995). In the second scenario, the total amount of standing genetic variation maintained includes local adaptation to a steep environmental gradient along one dimension of the species range. Here, the maintenance of additional standing genetic variation across the species range is effectively hidden when populations are equally well adapted to local environmental conditions, maintaining both patch occupancy and density.

Since the “gene‐swamping” effects arising from an environmental gradient ultimately limit a species potential range size, we were able to determine an effective upper limit to the amount of standing genetic variation that could be maintained by local adaptation within a given landscape. To do so, we measured various key population variables as emergent properties of a given landscape and species life history in response to an incremental increase in the environmental gradient *b*. These calculations were conducted after a burn‐in period of 20,000 generations once mutation–migration–selection balance had been established (see Figure S2). Range size started to contract once *b* exceeded an *effective* environmental gradient of 0.6 $\theta {\overset{¯}{d}}^{-1}\omega^{-1}$ (standardized to the species environmental tolerance and dispersal rate), though a decline in total population size was already evident once *b* exceeded 0.4 $\theta {\overset{¯}{d}}^{-1}\omega^{-1}$. Here, maladaptation at the range edge is evident at the deme level in terms of relatively lower $Q_{\text{ST}}:F_{\text{ST}}$ ratios and a lower mean heterozygosity (data not shown), a predicted consequence of “gene‐swamping” effects. Accordingly, we chose to use an environmental gradient of b = 3 $\theta {\overset{¯}{d}}^{-1}$ for our “steep environmental gradient” scenario, which equated to 0.3 $\theta {\overset{¯}{d}}^{-1}\omega^{-1}$ for a typical species (i.e. ω=10).

**Figure 1:**
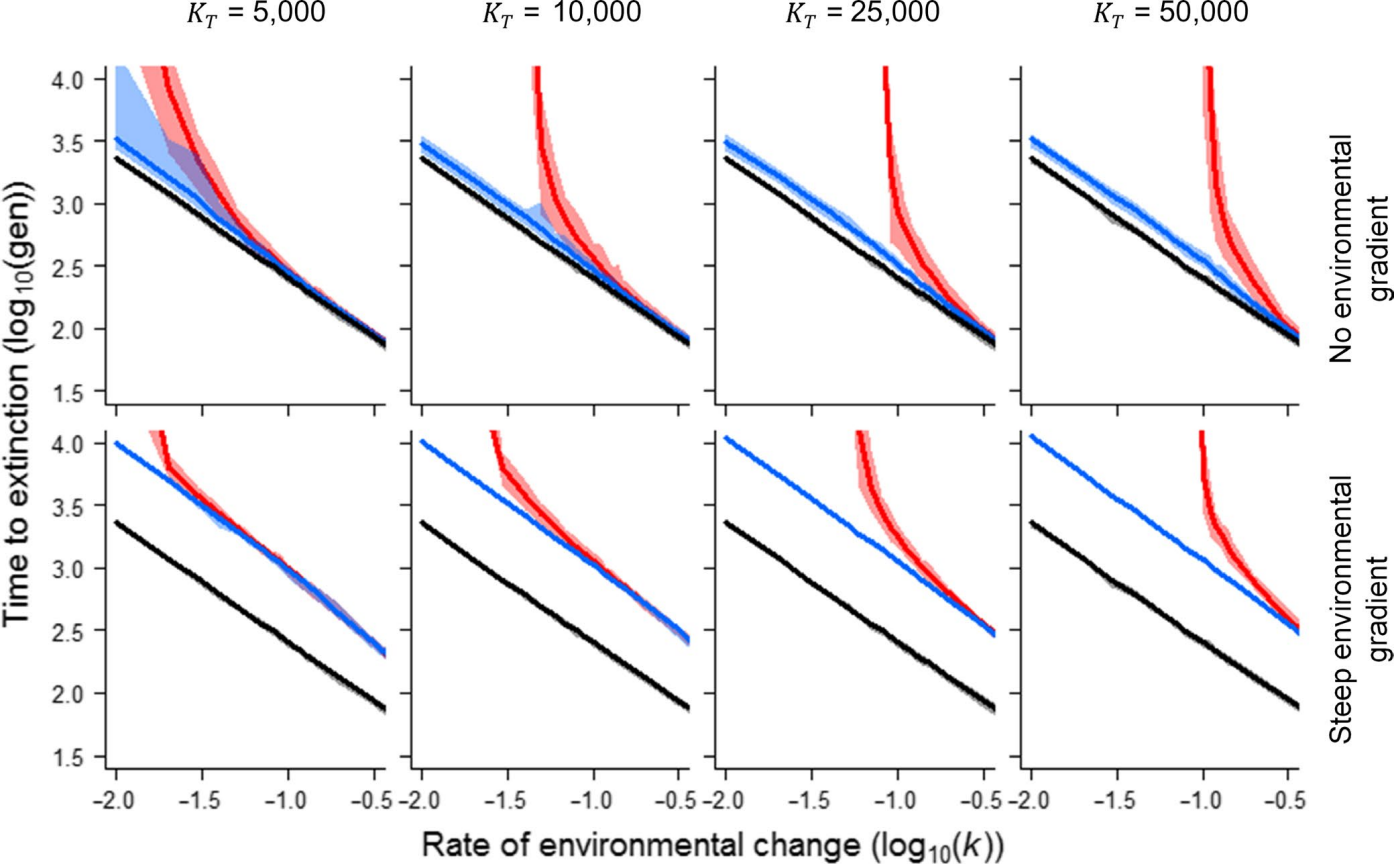
Influence of evolutionary adaptation on time to extinction in response to rate of environmental change demonstrated for a range of global carrying capacities ($K_{T}$). Model I (red lines) represents the full evolutionary response arising from both standing genetic variation and de novo mutation; Model II (blue lines) represents the adaptive response based solely on standing genetic variation; and Model III (black lines) represents the null model, where there was no heritable basis for variation in the mode for the environmental tolerance function. In the upper panels, a low level of standing genetic variation is associated with local adaptation to a single environmental mean; in the lower panels, a high level of standing genetic variation emerges as the consequence of local adaptation to a steep environmental gradient that is associated with the driver of directional selection (*b* = 0.3 $\theta {\overset{¯}{d}}^{-1}\omega^{-1}$; see text). Shadow bars indicate the minimum and maximum values based on 100 simulations per parameter combination for a single landscape replicate. Parameter values are *ω* = 10, $B_{\text{opt}}$ = 10, *c* = 1, *m* = 10

Figure 1 demonstrates the extent to which an evolutionary response could delay time to extinction under directional selection for a range of global carrying capacities and rates of environmental change. To understand the relative importance of standing genetic variation per se, we compare two alternative models of adaptation, one based solely on standing genetic variation versus another based on the combined contribution of both standing and de novo (mutational) variation (the full evolutionary response). We then compare these two models under the two extreme scenarios where the amounts of standing genetic variation maintained by local adaptation vary from low to high based on the steepness of the environmental gradient across the species range. As expected, high levels of standing genetic variation associated with local adaptation to a steep environmental gradient delay time to extinction, particularly at high rates of environmental change when mutation can only make a limited contribution.

**Figure 2:**
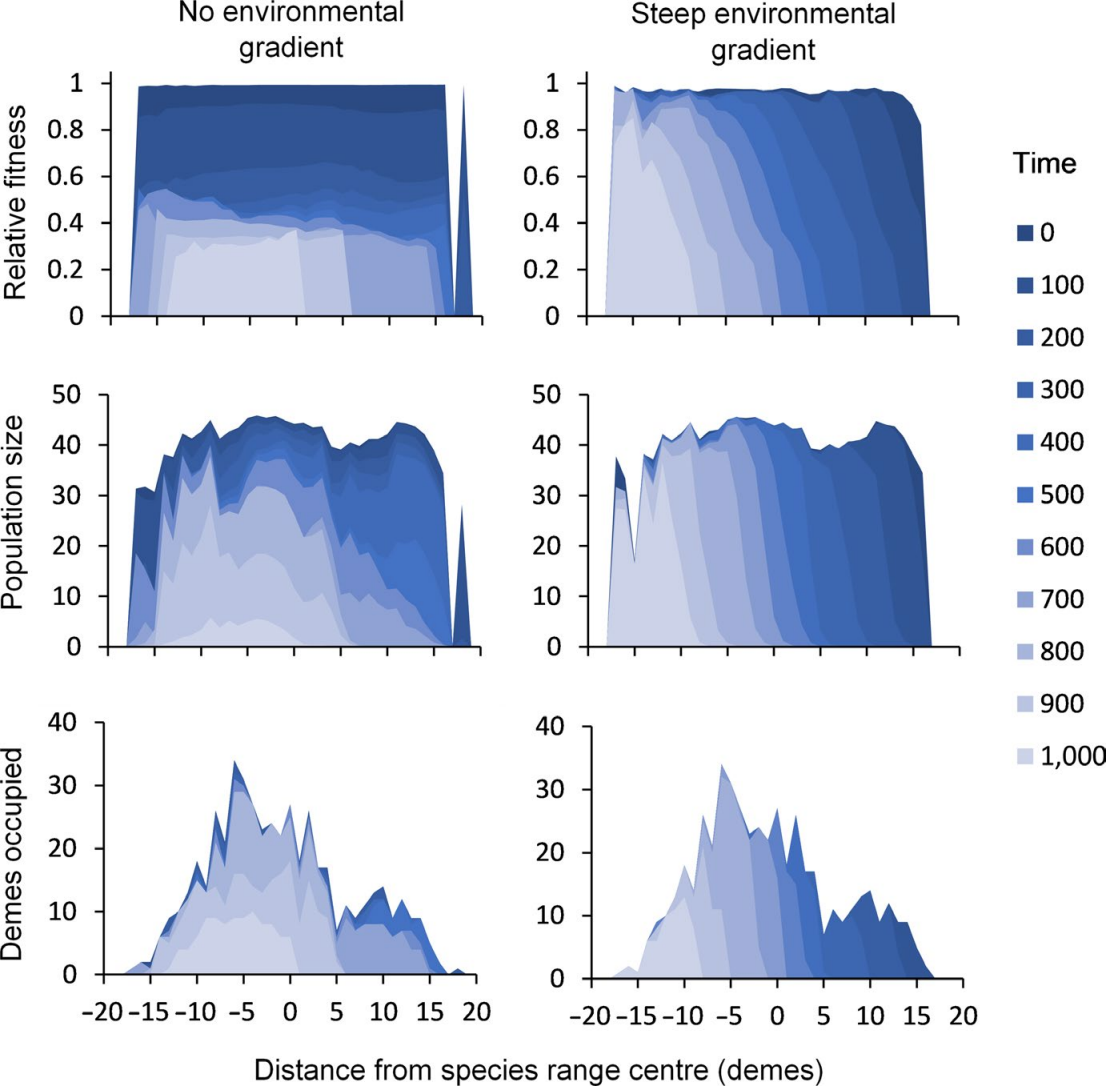
Example of extinction dynamics unfolding across space and time (generations) for a given rate of environmental change close to the critical rate (*k* = 0.1). When a species is locally adapted to a single environmental mean, directional selection induced by environmental change has the effect of reducing fitness across its entire range, with reductions in population size and habitat occupancy only manifesting close to the time of extinction (left panels). Note “relative fitness” here refers to the proportion of the maximum fitness value that could be attained in a local deme, i.e. when *z* =* θ*), Conversely, when a species is locally adapted to an environmental gradient associated with the driver of selection there is instead a steady travelling wave of declining adaptation, population size and habitat occupancy that starts at the retreating range edge (right panels). Response variables are mean values for demes. Parameter values: $K_{T}=25000$, *ω* = 10, $B_{\text{opt}}$ = 10, *c* = 1, *m* = 10

On closer examination of the population dynamics underlying extinction across the species range, we find distinct differences in how maladaptation manifests between the two alternative scenarios (Figure 2). For species adapted to a single environmental mean, environmental change causes an immediate loss in absolute fitness right across the species range. However, due to density dependence, resulting maladaptation fails to impact population size; it is not until the species distribution rapidly contracts to its core and extinction is imminent that maladaptation may be evident. In contrast, when a species is locally adapted to a steep environmental gradient associated with the driver of selection, maladaptation appears as a travelling wave of population extirpations that begins at the retreating range edge. In this latter scenario, the absolute fitness of individuals in populations towards the leading edge of the range is maintained by the gene flow of “preadapted” alleles arriving from populations situated closer to the retreating range edge. At the retreating range edge, however, migration is only likely to bring maladapted alleles any new beneficial mutations must emerge from a smaller effective population size (and number of haplotypes under the “continuum‐of‐allele” model).

While the relative contribution of standing genetic variation to rate of adaptation is lower at slow rates of environmental change when input from mutation is higher, it is not immediately apparent why the gene flow of preadapted alleles along an environmental gradient should lower the critical rate of change, *k_c_*, that is impede adaptation. The presence of “preadapted” alleles across the species range offers a clear short‐term adaptive advantage, particularly for those populations situated towards the leading edge of the range. However, as a consequence of the travelling wave of extirpations that occurs along an environmental gradient, a bottleneck emerges, which lowers the effective population size and limits the source of new mutations to perpetuate an adaptive response (Figure S3).

**Figure 3:**
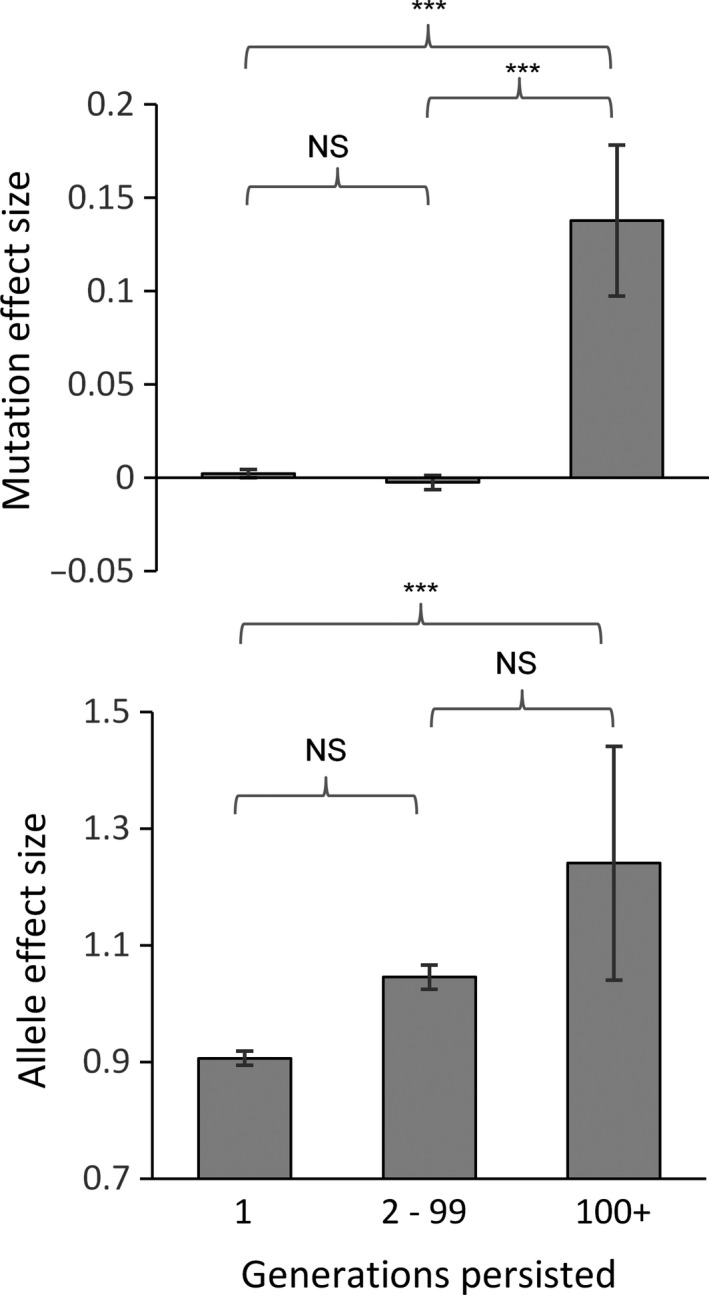
The mutants that persist longest within the species range under directional selection tend to be larger than average and are associated with a larger allele effect size

To gain a better understanding of how new mutations contribute to the adaptive response across the species range, we also followed the fate of every mutation arising in one typical simulation in which a species already adapted to a steep environmental gradient was subject to environmental change close to its critical rate ($K_{T}=10000$, *H* = 0.5, *ω* = 10, $B_{\text{opt}}$ = 10, *c* = 1, *m* = 10, k=0.1). In this single simulation, we recorded more than 70 thousand de novo mutations arising between the time environmental change started and species extinction. Very few of these mutations persisted under directional selection for more than 100 generations (0.25% of the total). Of those that did mutation, effect size was notably larger, averaging 0.58 versus 0.01 and −0.01 for those that persisted for either a single generation or between 2 and 99 generations, respectively (F_2,70,047_ = 7.718, *p* < .001). They were also associated with an allele effect size that was 35% larger on average (F_2,70,047_ = 17.96, *p* < .001) (Figure 3). Nonetheless, mutation and allele effect sizes appeared to have little to no influence on allele frequency, which was only weakly associated with time and location of the mutation's origin (Table 2). Specifically, the mutations that increased most in their frequency over 100 generations tended to be those which emerged later in the simulation and more towards the retreating range edge, when and where the influence of migrating alleles is expected to be weakest.

**Table 2:** The two most important factors determining the allele frequency of a de novo mutation over 100 generations following its emergence were as follows: (a) time, when during the simulation the mutation occurred and (b) location, where in the landscape with respect to the environmental gradient the mutation originated

Factor	Estimate	*t*‐value	*p*‐Value
(Intercept)	−2.440	−27.241	<.001
Time	3.786 × 10^−4^	1.899	.059
Location	−1.670 × 10^−2^	−2.991	.044
Allele effect size	1.928 × 10^−2^	3.701	.289
Mutation effect size	3.598 × 10^−2^	−0.855	.659

Note

Results of a full general linear model predicting log_10_(allele frequency) of 177 mutations: *F*_4,172_ = 4.597, *p* = .001, adjusted R‐squared = 0.076. Data were analysed using the glm function in R (version 3.5.0; R Development Core Team, 2018).

**Figure 4:**
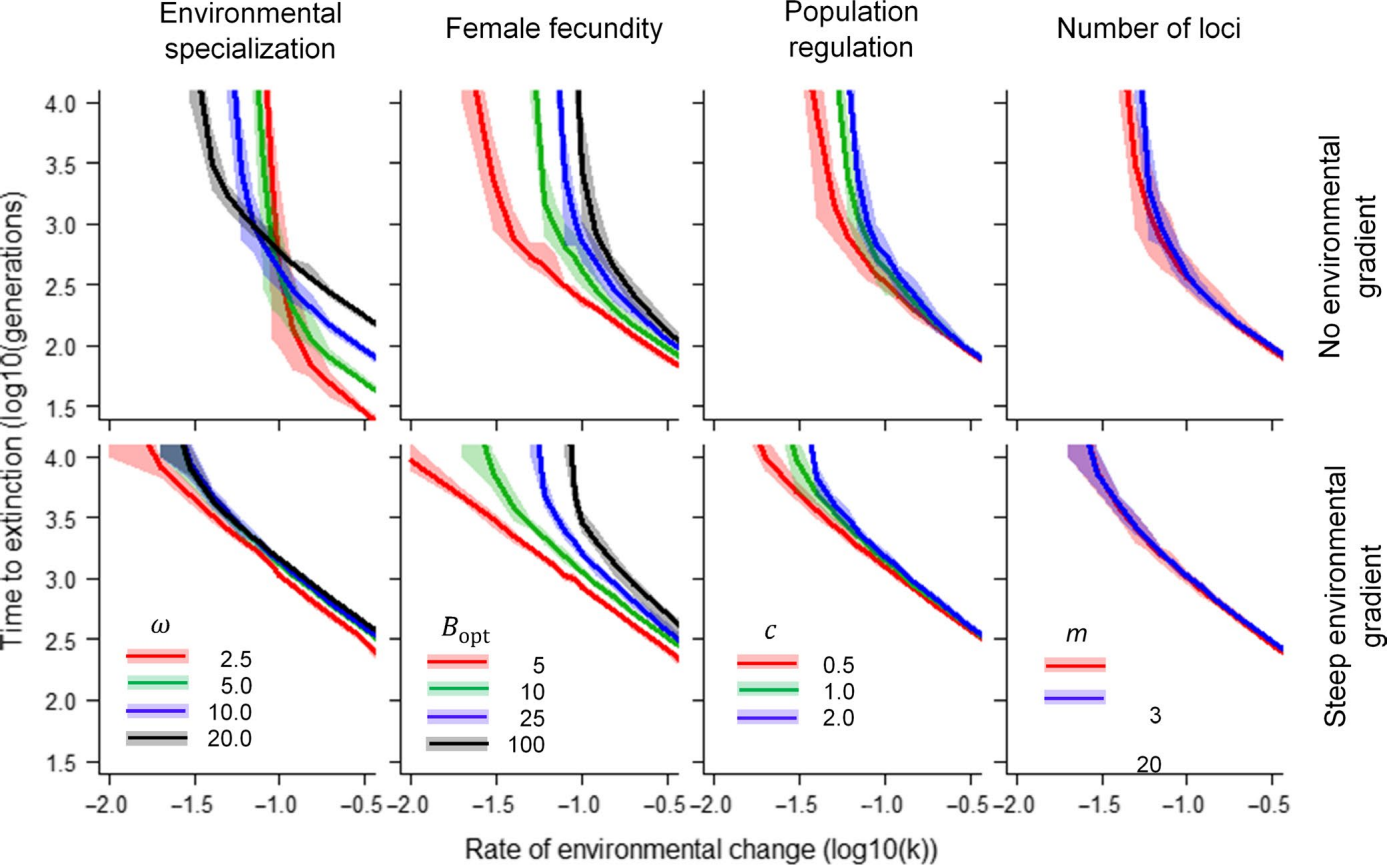
Consequences of local adaptation to an environmental gradient on predicted time to extinction under directional selection for various physiological, demographic and genetic scenarios in a given landscape replicate (Hurst = 0.5) and at a given carrying capacity ($K_{T}$ = 10,000). Note that “environmental specialization” is inversely related to the width of the Gaussian environmental tolerance function, $\omega^{2}$, such that a low value equates to a specialist. “Female fecundity” is implemented as a Poisson function, where $B_{\text{opt}}$ represents the maximum mean fecundity expected when the mode of the environmental tolerance function matches the local environment. “Population regulation” relates to the form of intraspecific competition, where *c* = 1 refers to logistic population growth, and “number of loci” assumes a constant genetic variance in z by scaling allelic effects accordingly. Unless otherwise stated, parameter values are *ω* = 10, $B_{\text{opt}}$ = 10, *c* = 1, *m* = 10

The issue of whether or not a species shows local adaptation to an environmental gradient may critically depend on interactions between its life history and the genetics underpinning adaptation. Figure 4 demonstrates diverse effects for four notable traits predicted to affect risk of extinction, namely environmental specialization (the inverse width of the environmental tolerance curve), reproductive output, the form of density‐dependent population regulation and the number of genetic loci underlying local adaptation.

### 3.1 Environmental specialization

Theoretical models predict that higher selection efficiency associated with environmental specialization (a narrower environmental tolerance curve) should increase the critical rate of environmental change but shorten time to extinction at higher rates of environmental change relative to a generalist strategy. However, this interaction between environmental specialization and the rate of environmental change is here observed to break down at the species level when subpopulations are locally adapted to a steep environmental gradient. In such cases, generalist strategies retain a marginal advantage irrespective of the rate of environmental change.

### 3.2 Reproductive output

Higher female fecundity provides greater opportunities for novel mutations to arise and contribute to adaptation under directional selection. This relative advantage appears to be amplified when populations are locally adapted to a steep environmental gradient, suggesting the negative effects of “gene swamping” may impact less fecund individuals to a greater extent.

### 3.3 Density‐dependent population regulation

Over‐compensatory population regulation (*c* > 1) is associated with a higher population growth rate that can increase extinction risk by causing oscillatory and even chaotic dynamics. However, under directional selection a relatively higher population growth rate extends time to extinction irrespective of rate of environmental change and whether or not there is local adaptation to a steep environmental gradient because it can help to maintain population size.

### 3.4 Number of loci

When genetic variance for *z* comprises many loci of small effect versus few loci of large effect, a species possesses a small advantage in tolerating a marginally higher mean critical rate of change. Since mutational variance was held constant, we attribute this result to the lower variance in time to extinction among simulations, a factor which is less important in the more complex scenarios when there is local adaptation to a steep environmental gradient.

**Figure 5:**
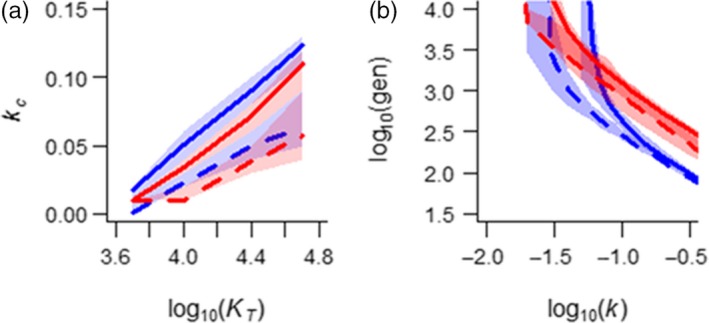
Populations that are locally adapted to fragmented landscapes (fully fragmented, *H* = 0.0, dashed lines vs. contiguous *H* = 1.0, solid lines) consistently (a) show lower critical rates of environmental change, $k_{c}$, at which extinction becomes inevitable under directional selection irrespective of the total landscape carrying capacity, $K_{T}$, and B) have shorter times to extinction at rates that exceed the critical rate $k_{c}$ (illustrated for $K_{T}=10,000$). Results shown for both a species locally adapted to a single environmental mean (blue) versus a species locally adapted to a steep environmental gradient (red). Parameter values are *ω* = 10, $B_{\text{opt}}$ = 10, *c* = 1, *m* = 10

**Figure 6:**
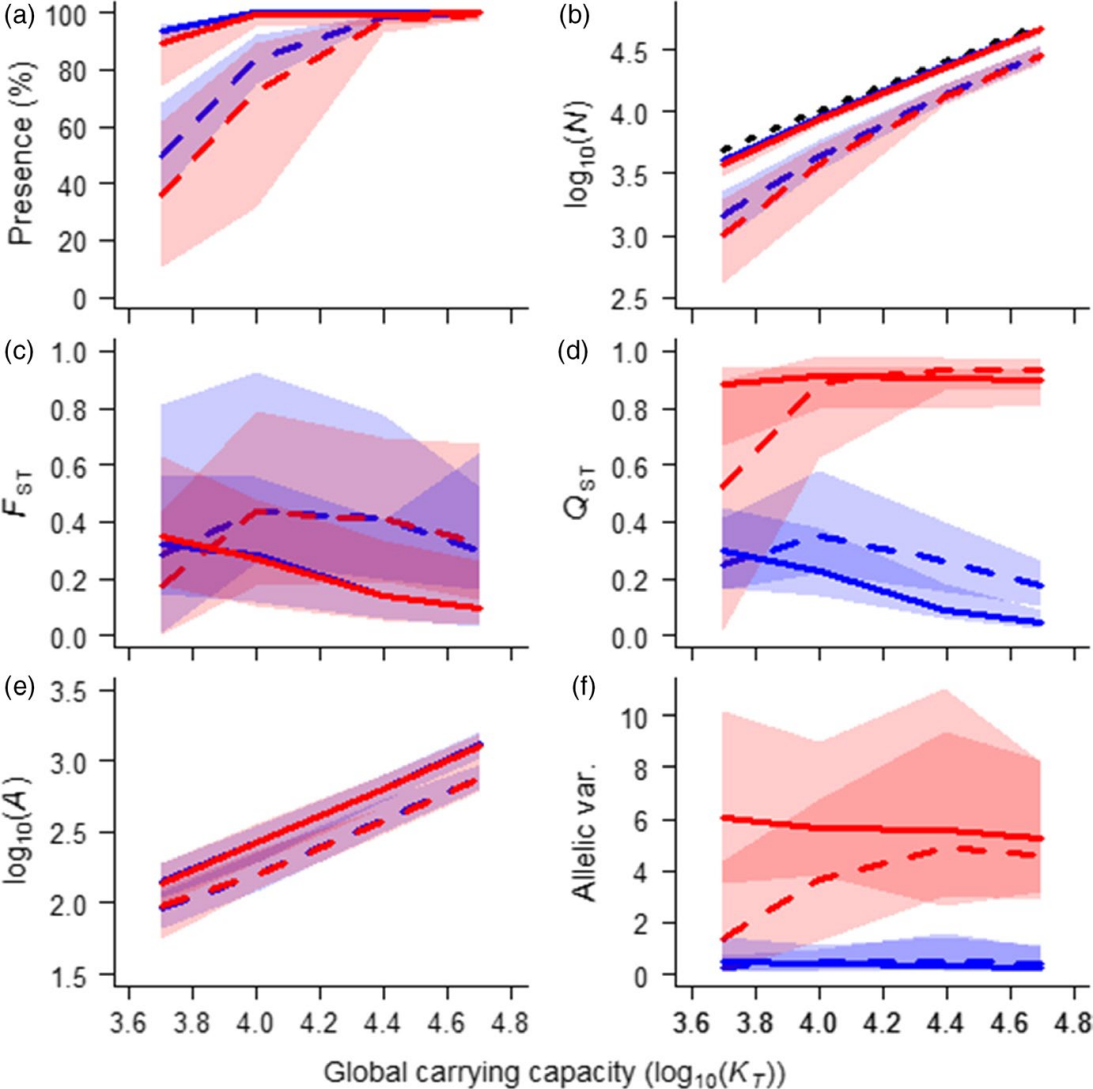
Demographic and genetic consequences of adaptation to landscapes varying in level of habitat fragmentation (fully fragmented, *H* = 0.0, dashed lines vs. contiguous *H* = 1.0, solid lines; dotted black line is the 1:1 reference line) as a function of global carrying capacity and whether the species was locally adapted to a single environmental men (blue) or locally adapted to a steep environmental gradient (red). Habitat fragmentation decreases (a) habitat occupation and (b) population size, and increases levels of (c) neutral genetic differentiation, particularly at low carrying capacities. At low carrying capacity, habitat fragmentation lowers genetic variance when populations are locally adapted to a steep environmental gradient but increases it for a species adapted to a single environmental mean. Adaptation to a fragmented landscape lowers (e) allelic richness and (f) allelic variance across the species range, though the latter was only evident at low carrying capacities when populations are locally adapted to a steep environmental gradient. Although habitat fragmentation and configuration yield substantial variation in genetic differentiation and local adaptation among simulations, persistence times when rate of environmental change is high are remarkably consistent. Parameter values are *ω* = 10, $B_{\text{opt}}$ = 10, *c* = 1, *m* = 10

“Preadapted” alleles can contribute greatly to rate of adaptation and persistence time but only if they are able to disperse across the species range. Habitat fragmentation in our virtual landscapes (Figure S1) lowered the critical rate change, particularly so for large populations (Figure 5a) though it had relatively little effect on persistence time at higher rates of environmental change (Figure 5b). This suggests that while poor habitat connectivity limits the extent to which de novo mutations can contribute to the *rate* of adaptation under directional selection due to a smaller effective population size (Figure 6a,b), it does not necessarily limit the *extent* of local adaptation along an environmental gradient and thus the range of preadapted alleles potentially available to facilitate a short‐term adaptive response. This may explain how habitat fragmentation and landscape configuration can greatly influence genetic differentiation among demes due to drift (Figure 6c) and the degree of selection efficiency (Figure 6d,f), and yet have only a modest influence on persistence time (Figure 5).

## 4 DISCUSSION

In our simulations, pre‐existing local adaptation to a steep environmental gradient versus a single environmental mean led to a ca. 2.8‐fold increase in time to extinction, with the greatest relative advantage evident at the highest rates of environmental change (Figure 1). This increase in persistence time was possible due to the greater amount of genetic variance maintained by migration–mutation–selection–drift (MMSD) balance across the species range. The steeper the environmental gradient, the greater the amount of genetic variation that can be maintained, even where this leads to a smaller potential range size and consequently total population size (Figure S2). Corollary increases in genetic variation may also occur at a smaller spatial scale within the landscape due to environmental heterogeneity (McDonald & Yeaman, 2018). When this local adaptation is ignored or such sources of additional genetic variation are effectively hidden (Paaby & Rockman, 2014), extinction risk under environmental change is likely to be overestimated. The presence of “preadapted” alleles allows for a more persistent adaptive response over time by providing additional “substrate” for selection. Moreover, the simple reassortment of alleles among ecotypes across the landscape mediated by gene flow ensures that at least some populations minimize maladaptation in the process. Understanding the spatial distribution of genetic variation across the species range, and the extent to which such preadapted alleles are capable of spreading across it, is thus pivotal to the development of predictive models of adaptation and extinction under environmental change (Chevin, Lande, & Mace, 2010), particularly where rates of change exceed the potential for novel mutations to sustain an adequate evolutionary response (IPCC, 2014; Maclean & Wilson, 2011).

Forecasting extinction risk is an inherently difficult task. The pragmatic approach in conservation biology is to recommend a minimum population size to hedge against the risk of population decline and loss of evolutionary potential due to stochastic processes (Traill, Brook, Frankham, & Bradshaw, 2010). Under directional selection, however, time to extinction becomes largely deterministic and a function of environmental tolerance, life history and trait heritability (Bürger & Krall, 2004; Lynch & Lande, 1993; Walters et al., 2012). Here, analytical models of adaptation can provide a simple metric of extinction risk—the critical rate of change *k_c_*. This is defined as the rate of environmental change at which the lag load under directional selection lowers population growth rate to 1, beyond which extinction becomes inevitable. Provided that the rate of environmental change does not exceed *k_c_*, the rate of adaptation should be sufficient to avoid extinction. However, our spatially explicit simulations highlight two important issues associated with its practical use. First, the spread of preadapted alleles across the landscape alone has the potential to enable populations to persist for hundreds and even thousands of generations longer than otherwise expected (Figure 1), long enough perhaps to avoid extinction since the extent of environmental change will be limited. Second, the spread of preadapted alleles across the species range has the potential to either accentuate or negate the predicted impact of a given life history, notably the breadth of environmental tolerance that can reflect the level of environmental specialization of the organism (Figure 4).

Clearly, the environmental tolerance of a species has important consequences for its predicted response to environmental change and much work has focussed on studying its implications. Perhaps the most notable suggestion is that tropical species are at greater risk of extinction than temperate ones due to their lower warming tolerance (Deutsch et al., 2008; Sunday, Bates, & Dulvy, 2011; Tewksbury, Huey, & Deutsch, 2008). As environmental specialists, tropical species moreover ought to also possess less genetic variance in their thermal optima to facilitate an adaptive response, and though a shorter generation time in the warmer tropics is predicted to help compensate for this loss in evolutionary potential (Walters et al., 2012), the spread of preadapted alleles among locally adapted populations would prove to be far more effective. The issue of extinction risk and potential for evolution under climate change remains hotly debated (Chown et al., 2010; Mitchell, Sgrò, & Hoffmann, 2011; Sgrò et al., 2010), particularly with respect to risk posed to those living in the biodiverse tropics (Hoffmann, 2010; Perez, Stroud, & Feeley, 2016; van de Pol, Jenouvrier, Cornelissen, & Visser, 2017). Nonetheless, recent studies suggest existing local adaptations will likely play a key role in reducing extinction risk by facilitating an adaptive response, for example, by reducing metabolic costs in ocean fish (Moffett, Fryxell, Palkovacs, Kinnison, & Simon, 2018) and by reducing bleaching events in corals (Matz, Treml, Aglyamova, & Bay, 2018). Detailed biological knowledge is needed to parameterize such complex models but where is not feasible our approach of simulating genetic variance from first principles as an emergent property of species life history and population size could serve as a useful ecological forecasting tool to investigate the potential for existing local adaptations to contribute to an adaptive response.

There is increasing recognition of the role evolution plays in conservation biology (Bürger & Krall, 2004; Stockwell, Hendry, & Kinnison, 2003) and the importance of eco‐evolutionary dynamics in determining responses to environmental change (Pelletier, Garant, & Hendry, 2009). Arguably, there is a disproportionate focus on the fate of small populations and management strategies to maintain their genetic diversity as the rapid rates of environmental change associated with anthropogenic causes pose a risk to most species, irrespective of their population size (Hoffmann & Sgrò, 2011; Walters et al., 2012). While the results of our spatially explicit simulations suggest forecasting extinction risk may be more challenging than proposed (Pearson et al., 2014), the use of mechanistic models provides an opportunity to better understand extinction debt and predict the consequences of dispersal (Gallagher, Hammerschlag, Cooke, Costa, & Irschick, 2015; Urban, 2015). A key finding from our simulations worthy of further empirical study is that gene flow among locally adapted populations can obfuscate the predicted relationship between the level of maladaptation and persistence time under directional selection.

Maladaptation arises from the mismatch between the mode of the individual's fitness function and its local environmental conditions, and it is the extent of this mismatch that determines a loss in fitness, which is used to calculate the critical rate of change *k_c_* under directional selection (Lynch & Lande, 1993). In our spatially explicit model, preadapted alleles from the range rear edge permit populations towards the leading edge to adapt faster, and maintain fitness for longer, while populations close to the rear edge adapt much more slowly since they are primarily dependent upon local standing genetic variation and de novo mutations arising in situ (under the continuum‐of‐allele model; Figure 2). The travelling wave of sequential maladaptation and extirpation predicted to emerge across the species range under directional selection offers new opportunities to study and predict time to extinction in a more precise way (Biktashev & Tsyganov, 2009; Garvie & Golinski, 2010). Our finding that migration of alleles permits some populations to maintain *absolute* fitness in at least part of the range also has important ramifications for ecological forecasting since any change in the *relative* fitness between competitors, predator and prey or disease and host is expected to impact local dynamics and range limits (Johansson, 2008; Sexton, McIntyre, Angert, & Rice, 2009; Urban, Scarpa, Travis, & Bocedi, 2019). For instance, the predicted consequence of the *relative* success of a species with abundant genetic diversity under directional selection is the accelerated demise of a less genetically diverse competitor. While it is recognized that an understanding of ecological interactions is necessary to predict the full impact of climate change on community structure (Cahill et al., 2013), our results suggest an understanding of the degree of local adaptation across a species range could in turn inform our understanding of the rate of species reassembly in terms of differential rates of adaptation.

To improve ecological forecasts, there is a need to better understand the extent of local adaptation across the range (Valladares et al., 2014) and to systemically monitor extirpation events across latitudinal or elevational clines (e.g. Sinervo et al., 2010). However, our simulation results suggest evaluating the extent of maladaptation per se is unlikely to provide a reliable indication of a species evolutionary potential, since evidence of a widespread loss in fitness could either reflect a lack of preadapted alleles available to facilitate an adaptive response or merely a current lack of gene flow among preadapted populations. Similarly, a lack of maladaptation could either reflect a lack of selection pressure or rapid adaptation enabled by the spread of preadapted alleles. In summary, what matters most to species persistence time is not the extent to which populations are or are not locally adapted, but the extent to which populations a) harbour preadapted alleles and b) these alleles can spread across the species range. The challenge for conservation biologists is to identify the source populations of these alleles and to maintain or assist gene flow among them.

Although genetic diversity is likely to be greatest towards the centre of a species range (Duncan, Crespi, Mattheus, & Rissler, 2015; Petit et al., 2003; Polechová & Barton, 2015), it is the contribution of favourable alleles from what otherwise are likely to be deemed genetically less diverse and maladapted populations towards the range edge that stand to make the greatest contribution to the rate of adaptation under directional selection, but these populations tend to be poorly studied (Hampe & Petit, 2005). While there is evidence that relic populations at the rear edge of the range can harbour greater amounts of genetic diversity (Hewitt, 1999), many appear to have played little to no substantial role in range expansion in response to past climate change events during the Holocene (de Bruyn, Hoelzel, Carvalho, & Hofreiter, 2011). The identification of suitable molecular markers for conservation purposes remains a challenge (Shafer et al., 2015), though we note here that whatever measure of adaptive genetic variance is used, there is very little variance in time to extinction as rates of environmental change exceed the critical rate (Figures 1 and 4). What matters here is the total amount of additive genetic variance present along an environmental gradient. When resources for conservation are limited (Ralls et al., 2018), the pragmatic approach could be to simply consider the steepness of an environmental gradient across a given range size as a proxy for evolutionary potential. In conservation management, range size is already identified as a key factor associated with species extinction risk (Lee & Jetz, 2011). Since local adaptation is considered ubiquitous knowing the what extent to which a species range extends across latitude or altitude, for example, could provide a useful indication of evolutionary potential with respect to climate warming (Angilletta, 2009; Berger, Postma, Blanckenhorn, & Walters, 2013).

An implicit assumption and possible limitation of our dynamic modelling approach is that populations are at migration–mutation–selection–drift balance prior to being subject to directional selection. In reality, habitat loss and fragmentation are expected to limit current gene flow, which would necessitate a management strategy to assist the migration of alleles via the creation of habitat corridors or through translocations. There has been much debate around such interventions and the value of preserving locally adapted genotypes versus preserving genetic diversity per se (McLachlan, Hellmann, & Schwartz, 2007) since such actions could have unforeseen and complex consequences for the ecology and evolution of a species (Hanski & Gaggiotti, 2004) and even the meta‐community (Urban et al., 2008). To avoid risks associated with selective sweeps (Latta, 2008), for example, any proposed translocation of genotypes would have to ensure a range of populations are represented by multiple individuals (McKay, Christian, Harrison, & Rice, 2005). There is also a possibility that populations could instead adapt to habitat fragmentation by changing dispersal rate or strategy (Cote et al., 2017; Saastamoinen et al., 2018), although whether this would increase or decrease gene flow across the species range would depend upon the extent of habitat isolation, and the associated costs and trade‐offs (Bonte et al., 2012; Gibbs, Saastamoinen, Coulon, & Stevens, 2010). What is clear is that local adaptation is ubiquitous and the potential impact of the resulting evolutionary load on rate of adaptation ought to be integrated into a predictive quantitative theory of extinction risk. The challenges for biological conservation will be to identify and manage these locally adapted populations and to reappraise the potential value of natural hybridization in maintaining genetic diversity and evolutionary potential in the wider gene pool (Currat, Ruedi, Petit, & Excoffier, 2008; Harris, Zhang, & Nielsen, 2019; Mable, 2013; Schmeller, Seitz, Crivelli, & Veith, 2005).

## CONFLICT OF INTEREST

None declared.

## Data Availability

The simulation data that support the findings of this study are openly available in Dryad Digital Repository at https://doi.org/10.5061/dryad.h7nj6qn (Walters, 2019; Walters & Berger, 2019)
