# Supplementary figures and images for: Implications of existing local (mal)adaptations for ecological forecasting under environmental change
Source: Evol Appl. 2019 Jul 30;12(7):1487–502. doi: 10.1111/eva.12840 (PMC6691230; doi:10.1111/eva.12840)

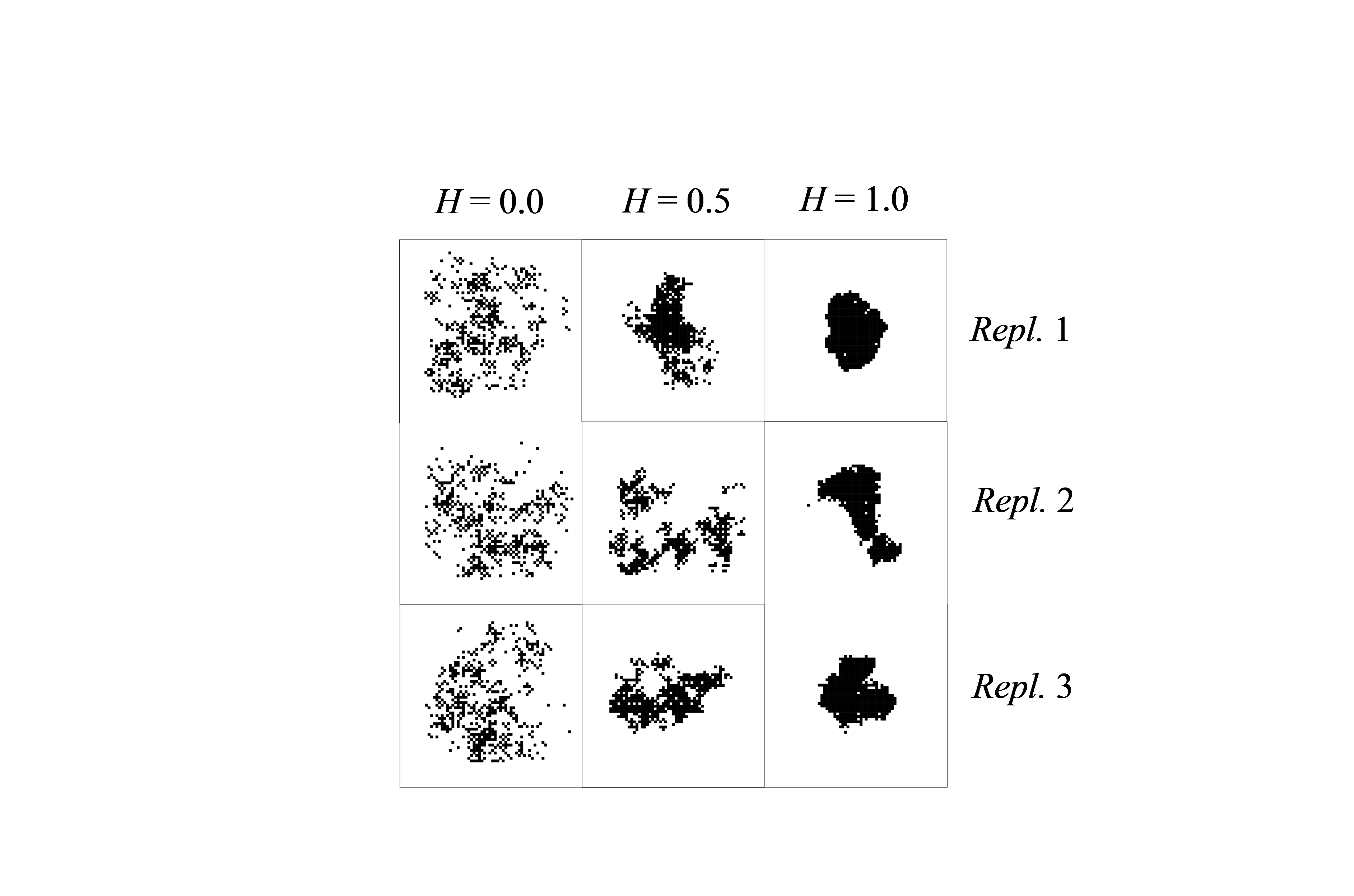

Supplement: Supplementary file 1 [file EVA-12-1487-s001.png]

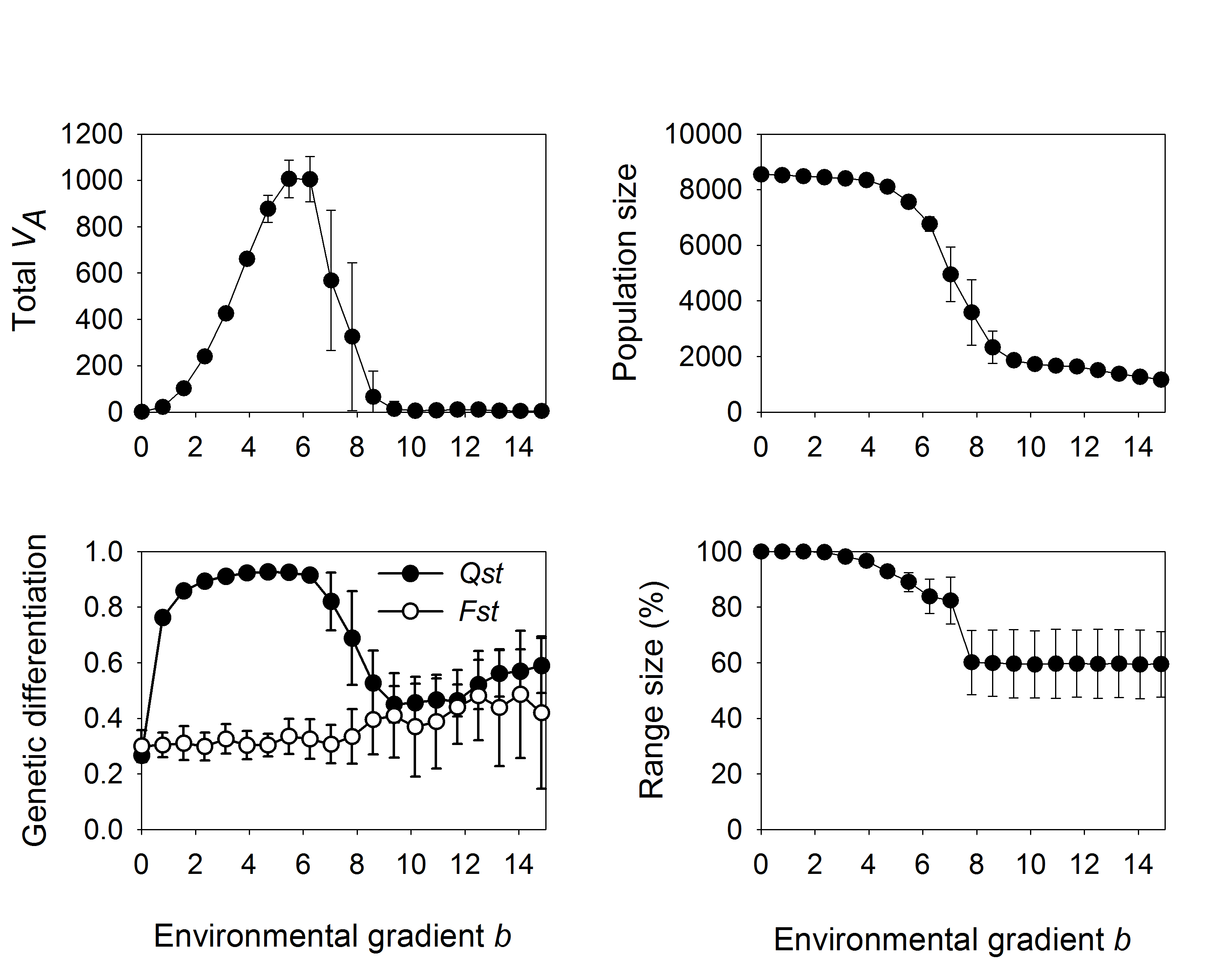

Supplement: Supplementary file 2 [file EVA-12-1487-s002.png]

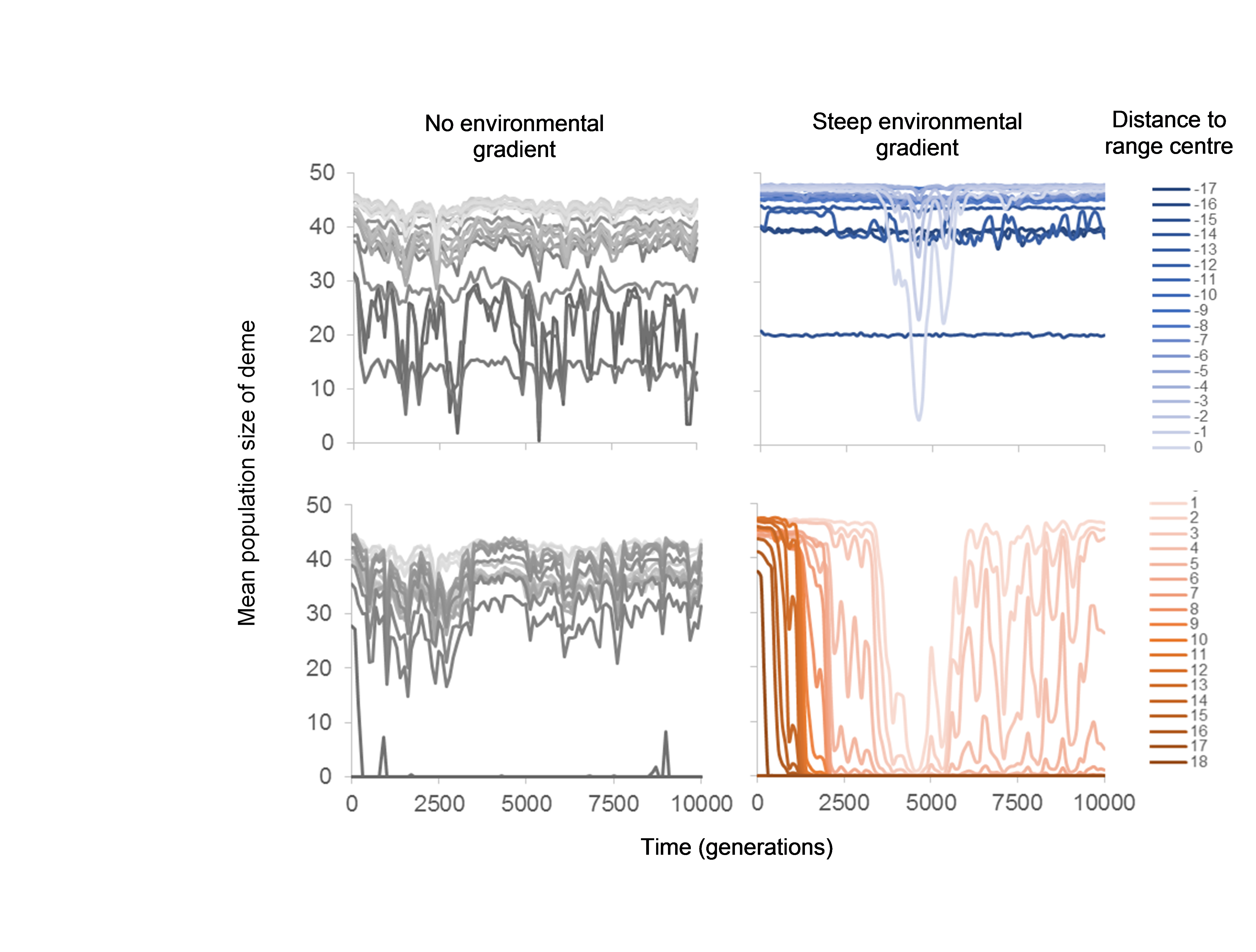

Supplement: Supplementary file 3 [file EVA-12-1487-s003.png]
